# Benzyne arylation of oxathiane glycosyl donors

**DOI:** 10.3762/bjoc.6.19

**Published:** 2010-02-22

**Authors:** Martin A Fascione, W Bruce Turnbull

**Affiliations:** 1 School of Chemistry, University of Leeds, Leeds, LS2 9JT, UK

**Keywords:** benzyne, 1,2-*cis*-glycosides, glycosyl acetates, oxathiane glycosyl donors, stereoselective glycosylations

## Abstract

The arylation of bicyclic oxathiane glycosyl donors has been achieved using benzyne generated in situ from 1-aminobenzotriazole (1-ABT) and lead tetraacetate. Following sulfur arylation, glycosylation of acetate ions proceeded with high levels of stereoselectivity to afford α-glycosyl acetates in a ‘one-pot’ reaction, even in the presence of alternative acceptor alcohols.

## Introduction

Carbohydrates play important roles in many biological processes including tumour metastasis [1–2], bacterial and viral recognition [3–5], and the immunological response [6–8]. In order to obtain pure samples of oligosaccharides for biological studies, carbohydrate chemists must overcome the myriad challenges presented by their complex synthesis. The most important challenge is control over the stereoselectivity of reactions at the anomeric centre; in particular for the stereoselective synthesis of 1,2-*cis*-glycosides [9]. This area has been the subject of much fervent study in the last two decades, and has led to many significant developments [10–11]. However, despite these advances, modern synthetic carbohydrate chemistry has still to provide a general method for the efficient synthesis of 1,2-*cis*-α-glycosidic linkages.

**Scheme 1:**
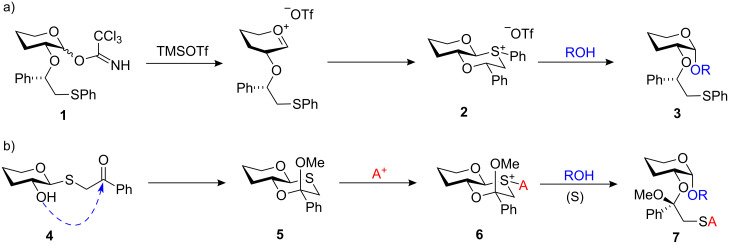
a) Boons’ chiral auxiliary-based approach to α-stereoselective glycosylations. b) Modified strategy for stereoselective glycosylations using oxathiane ketal glycosyl donors **5**.

In 2005 Boons and co-workers reported an elegant chiral auxiliary-based glycosylation protocol for the synthesis of 1,2-*cis*-α-glycosides [12]. Completely stereoselective glycosylation was achieved when a thiophenyl-containing chiral auxiliary was attached to *O*-2 of an imidate glycosyl donor **1** (Scheme 1a). Low temperature ^1^H NMR spectroscopy studies confirmed the formation of a quasi-stable *trans*-decalin intermediate **2**, which was able to cause glycosylation to take place from the α-face of the glycosyl donor. We sought to improve this strategy and recently reported a novel class of bicyclic oxathiane ketal donors **5** containing an inbuilt α-directing group (Scheme 1b) [13]. The principal objective of our approach was to develop a thioglycoside donor that could mimic the key *trans*-decalin intermediate **2** by using the sulfur-containing auxiliary as both the anomeric leaving group and α-directing participating group. An efficient synthesis of the key bicyclic intermediate was achieved starting from a simple thioglycoside **4** where the essential β-sulfur linkage was already installed, followed by a regio and stereoselective cyclisation onto the *O*-2 position to afford oxathiane glycosyl donor framework **5**. The oxathiane ketal donor **5** is then already pre-organised to give a 1,2-*cis* directing group upon activation, and afford 1,2-*cis*-glycosides **7** on alcohol addition.

Following the synthesis of the oxathiane ketal glycosyl donors **5**, activation of the β-thioglycoside linkage was necessary to form the key *trans*-decalin sulfonium ion **6**, and turn the α-directing participating group into an anomeric leaving group reactive enough to partake in glycosylations. Thioglycosides are widely used as glycosyl donors [14–15], and many different reagents are available for their activaton including *N*-iodosuccinimide (NIS)/TMSOTf [16], dimethyl(methylthio)sulfonium trifluoromethanesulfonate (DMTST) [17–18], PhSeOTf [19], MeS-SMe/Tf_2_O [20], and MeOTf [21–23]. However, in order to recreate the reactive sulfonium ion used by Boons, it would be necessary to activate the anomeric sulfur with a phenyl group. Herein we describe our synthetic endeavours to achieve this goal and the first use of benzyne as an activating agent for thioglycosides [24].

## Results and Discussion

**Scheme 2:**
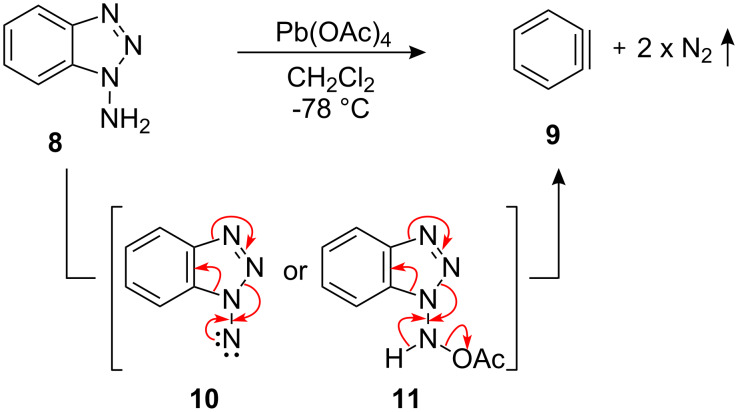
Benzyne generation from 1-ABT.

The method chosen for in situ benzyne (**9**) generation was the reaction of 1-aminobenzotriazole (1-ABT) (**8**) with lead tetraacetate by the procedure pioneered by Rees and co-workers (Scheme 2) [25–28]. The low reaction temperature was expected to be compatible with stereoselective glycosylation. Following the reaction of 1-ABT (**8**) with lead tetraacetate, benzyne (**9**) formation is believed to occur via degradation of either an *N*-nitrene intermediate **10** or an *N*-acetate substituted intermediate **11** the driving force for which is the release of di-nitrogen [29].

**Scheme 3:**
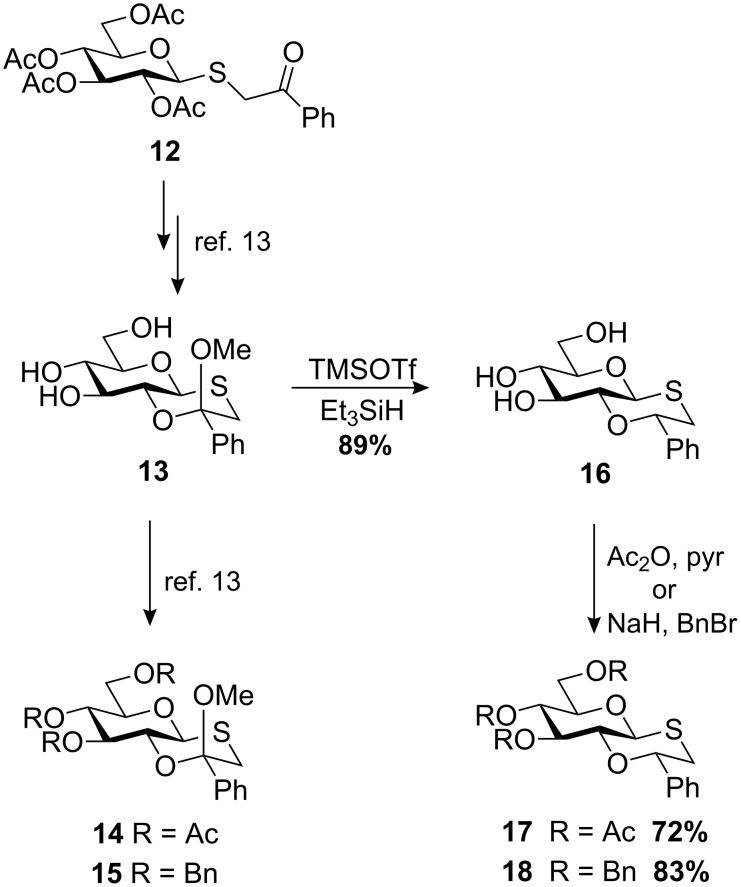
Oxathiane donor synthesis.

The synthesis of oxathiane ketal **13** was achieved in two steps from thioglycoside **12** as previously reported, followed by protection to afford the acetylated oxathiane ketal **14** or the benzylated oxathiane ketal **15** (Scheme 3) [13]. Ketal **13** was also reduced to the novel oxathiane ether **16** in 89% yield, and protected to afford acetylated oxathiane ether **17** and benzylated oxathiane ether **18** in yields of 72% and 83%, respectively.

**Scheme 4:**
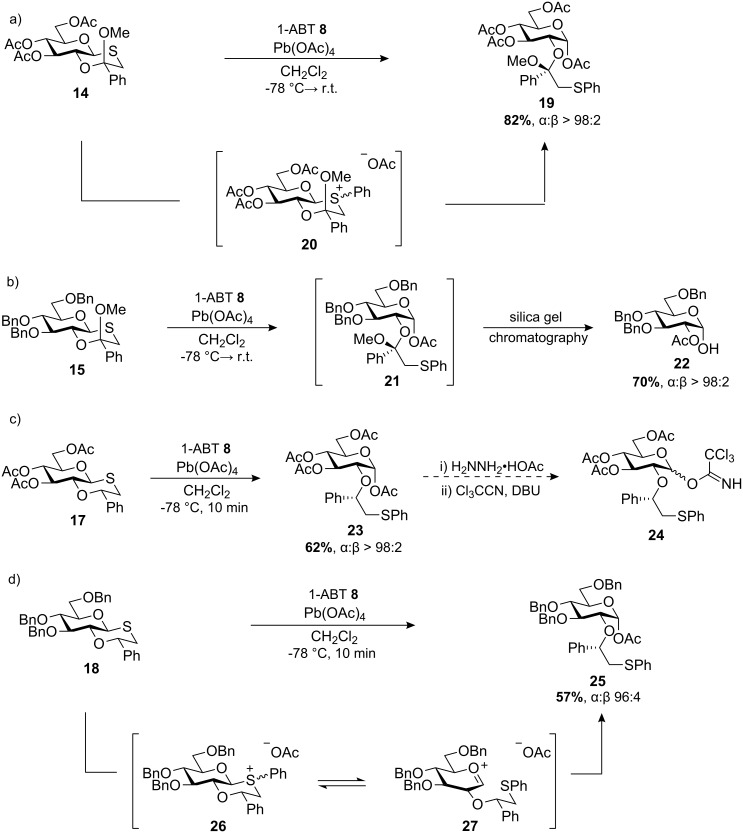
Arylation/acetate glycosylation of oxathiane glycosyl donors.

With the oxathiane ketal and ether donors in hand, initial studies focussed on benzyne arylation in the absence of any alcohol acceptor. Unexpectedly, when activated under the reaction conditions, acetylated oxathiane ketal **14** afforded α-glycosyl acetate **19** stereoselectively in 82% yield (Scheme 4a). Presumably, the mechanism proceeds via sulfur arylation with benzyne to afford putative phenyl sulfonium ion **20**, followed by glycosylation of the acetate anion. The very high α-stereoselectivity (α:β > 98:2) of the reaction was in line with selectivities previously observed for intermediate oxathiane sulfonium ions [13]. When the reaction was repeated in the absence of 1-ABT, the starting material was unchanged, thus precluding the possibility of initial sulfur activation by lead tetraacetate.

Arylation of benzylated oxathiane ketal **15** under identical conditions also afforded α-glycosyl acetate **21** as the sole crude product as evidenced by ^1^H NMR spectroscopy (Scheme 4b). However, only α-hemiacetal **22** was isolated in 70% yield following purification by flash silica chromatography [30]. Cleavage of the acyclic *O*-2 ketal on glycosyl acetate **21**, followed by acetyl transfer from *O*-1 to *O*-2 could account for this transformation [31].

Arylation/acetate glycosylation using oxathiane ether donor **17** also occurred readily to give the α-glycosyl acetate **23** in 62% yield with complete anomeric control (Scheme 4c). It is of interest to note that glycosyl acetate **23** has been reported previously by Boons and co-workers as an advanced intermediate in their synthesis of trichloroacetimidate donor **24** bearing a 2-*O*-(1*S*)-phenyl-2-(phenylsulfanyl)ethyl group (Scheme 4c) [12]. Although the benzyne arylation method does not allow us to access α-glycosides directly, it could be beneficial as an alternative route to glycosyl donors bearing the Boons’ participating group. This strategy has the advantage of utilising the inherent chirality of the sugar to determine the stereochemistry of the key benzylic centre in the chiral auxiliary, and also facilitates regioselective attachment of the auxiliary group to *O*-2.

The benzylated oxathiane ether **18** also afforded a glycosyl acetate **25** in 57% yield but on this occasion as a 96:4 (α:β) mixture of anomers (Scheme 4d). This slight drop in stereoselectivity is consistent with the increased reactivity of benzylated relative to acetylated thioglycoside donors [13,32], commonly attributed to greater stabilisation of the developing positive charge on an oxacarbenium intermediate **27** on the reaction pathway. Overall, reactions using the oxathiane ether donors proceeded more rapidly than those with the oxathiane ketal donors, indicating that the methoxy substituent moderates the reactivity of the glycosyl donors.

Attempts to intercept putative phenyl sulfonium ions such as **20** and **26** in glycosylation reactions with other acceptors prior to acetate glycosylation were in vain, presumably due to the high effective concentration of acetate anions in solution. Therefore, alternative oxidising agents for benzyne formation were also investigated in the hope that glycosylation with external alcohols would be easier to achieve if the phenyl sulfonium ion was formed with a less reactive counter ion. However, oxidation of 1-ABT in the presence of ketal **14** with NIS [33], or hypervalent iodine (III) with either bis(acetoxy)iodobenzene [PhI(OAc)_2_] [34] or bis(trifluoroacetoxy)iodobenzene [PhI(OCOCF_3_)_2_] were unsuccessful [35], resulting in at best only trace amounts of phenyl sulfonium ion formation. Under these reaction conditions, nitrogen evolution and presumably benzyne formation, was much slower than when using lead tetraacetate as the oxidising agent. Further studies using the more reactive Zefirov’s reagent (µ-oxobis[(trifluoromethanesulfonato)(phenyl)iodine]) [36–37] were also undertaken. Preliminary results were promising yielding simple α-glycosides and a full study will be reported in due course. Unfortunately, attempted extension of the arylation/acetate glycosylation methodology to conventional thiophenyl glycosyl donors was disappointing, as experiments either did not proceed to completion, or were hampered by oxidation of the thiophenyl group in the presence of lead tetraacetate [38].

## Conclusion

In conclusion, it has been demonstrated that benzyne arylation of novel oxathiane glycosyl donors can be achieved using a combination of 1-ABT and lead tetraacetate. Following arylation, glycosylation with an acetate anion takes place with a high degree of stereoselectivity to afford 1,2-*cis*-α-acetates.

## Supporting Information

Supporting Information File 1 features full experimental data for the synthesis of compounds **16–19**, **22**, **23** and **25**.

File 1 Experimental data for the synthesis of compounds **16–19**, **22**, **23** and **25**.
