# Supplementary material for: Benzyne arylation of oxathiane glycosyl donors
Source: Beilstein J Org Chem. 2010 Feb 22;6:19. doi: 10.3762/bjoc.6.19 (PMC2870982; doi:10.3762/bjoc.6.19)
Supplement: File 1 — Experimental data for the synthesis of compounds 16–19, 22, 23 and 25. [file Beilstein_J_Org_Chem-06-19-s001.pdf]

Supporting Information

for

Benzyne arylation of oxathiane glycosyl donors

Martin A. Fascione and W. Bruce Turnbull*

School of Chemistry, University of Leeds, Leeds, LS2 9JT, UK.

* Corresponding author

E-mail: W. Bruce Turnbull – w.b.turnbull@leeds.ac.uk

Experimental data for the synthesis of compounds 16-19, 22, 23 and 25.

Experimental

General Methods: All solvents were dried prior to use, according to standard methods [1]. Where appropriate anhydrous quality material was purchased. All solvents used for flash chromatography were GPR grade, except hexane and ethyl acetate, when HPLC grade was used. All concentrations were performed *in vacuo,* unless otherwise stated. All reactions were performed in oven dried glassware under a N_2(g) atmosphere, unless otherwise stated. ^{1}H NMR spectra were recorded at 500 MHz on a Bruker avance 500 instrument or at 300 MHz on a Bruker avance 300 instrument. ^{13}C NMR spectra were recorded at 75 MHz on a Bruker avance 300 instrument. Chemical shifts are given in parts per million downfield from tetramethylsilane. The following abbreviations are used in ^{1}H NMR analysis: s = singlet, d = doublet, t = triplet, q = quartet, m = multiplet, dd = double doublet, dt = double triplet, td = triple doublet, ddd = double double doublet. Electrospray (ES+) ionisation mass spectra were obtained on a Micromass LCT-KA111 mass spectrometer, and high resolution ES+ were performed on a Bruker Daltonics MicroTOF mass spectrometer. Melting points were obtained on a Reichert hot-stage apparatus and are uncorrected. Optical rotations were measured at the sodium D-line with an Optical Activity AA-1000 polarimeter. $[\alpha]_D$ values are given in units of 10^{-1} deg cm^2 g^{-1}. Analytical T.L.C. was performed on silica gel 60-F^{254} (Merck) with detection by fluorescence and/or charring following immersion in a 5% H_2SO_4/methanol solution, unless otherwise stated.

2-(*S*)-Phenyl-(1,2-dideoxy-β-D-glucopyranoso)[1,2-*e*]-1,4-oxathiane (16)

TMSOTf (2.56 mL, 14.2 mmol) was added dropwise to a solution of 2-methoxy-2-(*S*)-phenyl-(1,2-dideoxy-β-D-glucopyranoso)[1,2-*e*]-1,4-oxathiane **13** (3.11 g, 9.4 mmol) in $C_2H_4Cl_2$ (30 mL) at 0 °C, followed by addition of triethylsilane (2.29 mL, 14.2 mmol). After 1 h 25 min the reaction mixture was quenched with methanol (5 mL), and neutralised with triethylamine. The reaction mixture was concentrated to leave a colourless foam. The crude foam was purified by flash column

chromatography (silica; 96:4 (v/v) CH_2Cl_2-methanol→9:1 (v/v) CH_2Cl_2-methanol) to afford **16** (2.5 g, 89%) as a colourless foam; $[\alpha]_D^{23}$ +68.3 (*c* 1.2, $CHCl_3$); R_F 0.28 (9:1 (v/v) CH_2Cl_2-methanol); IR (ν_{max}/cm^{-1}): 3374 (OH); ^{1}H-NMR (500 MHz, $CDCl_3$); 7.38-7.32 (m, 5H, ArH), 4.69 (dd, 1H, $J_{PhCH,SCHax}$ 10.8 Hz, $J_{PhCH,SCHeq}$ 1.6 Hz, PhCH), 4.47 (d, 1H, $J_{1,2}$ 8.9 Hz, H-1), 3.94 (dd, 1H, $J_{6,6'}$ 12.0 Hz, $J_{5,6}$ 3.3 Hz, H-6), 3.83 (dd, 1H, $J_{6,6'}$ 12.0 Hz, $J_{5,6'}$ 4.9 Hz, H-6'), 3.73 (dd, 1H, $J_{2,3}$ 9.2 Hz, $J_{3,4}$ 9.0 Hz, H-3), 3.67 (dd, 1H, $J_{3,4}$ 9.0 Hz, $J_{4,5}$ 8.9 Hz, H-4), 3.55 (m, 1H, H-5), 3.53 (dd, 1H, $J_{2,3}$ 9.2 Hz, $J_{1,2}$ 8.9 Hz, H-2), 3.06 (dd, 1H, $J_{SCHax\text{-}eq}$ 14.1 Hz, $J_{PhCH,SCHax}$ 10.8 Hz, SCH_{ax}), 2.82 (br s, 1H, OH), 2.75 (dd, 1H, $J_{SCHax\text{-}eq}$ 14.1 Hz, $J_{PhCH,SCHeq}$ 1.6 Hz, SCH_{eq}), 2.09 (br s, 1H, OH), 1.59 (br s, 1H, OH); ^{13}C-NMR (75 MHz, $CDCl_3$); 140.1, 128.8, 128.6, 126.0 (ArC), 83.3 (C-2), 80.4 (C-5), 80.2 (PhCH), 75.5 (C-1), 75.4 (C-3), 70.6 (C-4), 62.3 (C-6), 35.4 (SCH_2); HRMS: Found $[M+Na]^+$ 321.0772, $C_{14}H_{18}O_5SNa$ requires 321.0767.

2-(*S*)-Phenyl-(3,4,6-tri-*O*-acetyl-1,2-dideoxy-β-D-glucopyranoso)[1,2-*e*]-1,4-oxathiane (17)

Acetic anhydride (1.05 mL, 11.07 mmol) was added to a solution of 2-(*S*)-phenyl-(1,2-dideoxy-β-D-glucopyranoso)[1,2-*e*]-1,4-oxathiane **16** (1 g, 3.35 mmol) in pyridine (10 mL), at 0 ºC. The reaction mixture was stirred, allowing the temperature to rise to r.t. After 14 h 30 min, the reaction mixture was concentrated. The residue was redissolved in CH_2Cl_2 (25 mL) and washed with 1M HCl (25 mL), aq. $NaHCO_3$ (25 mL) and aq. NaCl (25 mL). The organic phase was dried (Na_2SO_4) and concentrated to afford an orange foam. The crude foam was recrystallised from methanol to afford **17** (1.01 g, 72%) as colourless needles, m.p. 128.8-132.3 ºC; $[\alpha]_D^{25}$ −24.0 (*c* 0.45, $CHCl_3$); R_F 0.47 (1:1 (v/v) hexane-ethyl acetate); IR (ν_{max}/cm^{-1}): 1747 (C=O); ^{1}H-NMR (500 MHz, $CDCl_3$); 7.36-7.26 (m, 5H, ArH), 5.28 (dd, 1H, $J_{2,3}$ 9.5 Hz, $J_{3,4}$ 9.5 Hz, H-3), 5.15 (dd, 1H, $J_{3,4}$ 9.5 Hz, $J_{4,5}$ 9.7 Hz, H-4), 4.70 (dd, 1H, $J_{PhCH,SCHax}$ 10.6 Hz, $J_{PhCH,SCHeq}$ 1.7 Hz, PhC<u>H</u>), 4.51 (d, 1H, $J_{1,2}$ 9.0 Hz, H-1), 4.26 (dd, 1H, $J_{6,6'}$ 12.5 Hz, $J_{5,6'}$ 5.0 Hz, H-6'), 4.15 (dd, 1H, $J_{6,6'}$ 12.5 Hz, $J_{5,6'}$ 2.1 Hz, H-6), 3.83 (ddd, 1H, $J_{4,5}$ 9.7 Hz, $J_{5,6'}$ 5.0 Hz, $J_{5,6}$ 2.1 Hz, H-5), 3.75 (dd, 1H, $J_{1,2}$ 9.0 Hz, $J_{2,3}$ 9.5 Hz, H-2), 2.98 (dd, 1H, $J_{SCHax\text{-}eq}$ 14.1 Hz, $J_{PhCH,SCHax}$ 10.6 Hz, SCH_{ax}), 2.82 (dd, 1H, $J_{SCHax\text{-}eq}$ 14.1 Hz, $J_{PhCH,SCHeq}$ 1.7 Hz, SCH_{eq}),

2.10 (s, 3H, C(O)CH_3), 2.05 (s, 3H, C(O)CH_3), 2.00 (s, 3H, C(O)CH_3); ^{13}C-NMR (75 MHz, $CDCl_3$); 169.5 (C=O), 139.5, 128.5, 127.9, 125.3 (ArC), 81.0 (C-2), 79.5 (PhCH), 76.6 (C-1), 75.8 (C-5), 72.9 (C-3), 68.5 (C-4), 62.1 (C-6), 35.6 (SCH_2), 20.8, 20.7, 20.7 (C(O)$\underline{C}H_3$); HRMS: Found $[M+Na]^+$ 447.1069, $C_{20}H_{24}O_8SNa$ requires 447.1084.

2-(*S*)-Phenyl-(3,4,6-tri-*O*-benzyl-1,2-dideoxy-β-D-glucopyranoso)[1,2-*e*]-1,4-oxathiane (18)

Sodium hydride (60% dispersion in oil, 560 mg, 14 mmol) was added in portions to a solution of 2-(*S*)-phenyl-(1,2-dideoxy-β-D-glucopyranoso)[1,2-*e*]-1,4-oxathiane **16** (1.1 g, 3.69 mmol) in DMF (15 mL), at 0 ºC. The reaction mixture was stirred for 5 min before benzyl bromide (1.67 mL, 14 mmol) was added dropwise. The reaction mixture was stirred, allowing the temperature to rise to r.t. After 14 h 30 min, the reaction mixture was quenched with methanol (10 mL) and concentrated. The residue was redissolved in CH_2Cl_2 (30 mL), washed with aq. NaCl (2 x 30 mL), dried (Na_2SO_4) and concentrated to leave a yellow solid. The crude solid was purified by flash chromatography (silica; 3:1 hexane-ethyl acetate→1:1 (v/v) hexane-ethyl acetate) to afford **18** as colourless needles (1.74 g, 83%), m.p. 102.9-104.5 (from 1:1 (v/v) hexane-ethyl acetate); $[\alpha]_D^{23}$ +33.6 (*c* 1.14, $CHCl_3$); R_F 0.71 (1:1 (v/v) hexane-ethyl acetate); IR (ν_{max}/cm^{-1}): 3029 (C-H), 1090 (C-OR); ^{1}H-NMR (500 MHz, $CDCl_3$); 7.40-7.15 (m, 20H, ArH), 4.92 (d, 1H, *J* 11.2 Hz, $OC\underline{H}_2Ph$), 4.86 (d, 1H, *J* 10.9 Hz, $OC\underline{H}_2Ph$), 4.75 (dd, 1H, $J_{PhCH,SCHax}$ 10.6 Hz, $J_{PhCH,SCHeq}$ 1.7 Hz, $PhC\underline{H}$), 4.74 (d, 1H, *J* 11.2 Hz, $OC\underline{H}_2Ph$), 4.61 (d, 1H, *J* 12.1 Hz, $OC\underline{H}_2Ph$), 4.54 (d, 1H, *J* 10.9 Hz, $OC\underline{H}_2Ph$), 4.53 (d, 1H, *J* 12.1 Hz, $OC\underline{H}_2Ph$), 4.41 (d, 1H, $J_{1,2}$ 8.6 Hz, H-1), 3.79-3.70 (m, 5H, H-2, H-3, H-4, H-6, H-6'), 3.62 (m, 1H, H-5), 3.04 (dd, 1H, $J_{SCHax\text{-}eq}$ 14.0 Hz, $J_{PhCH,SCHax}$ 10.6 Hz, SCH_{ax}), 2.79 (dd, 1H, $J_{SCHax\text{-}eq}$ 14.0 Hz, $J_{PhCH,SCHeq}$ 1.7 Hz, SCH_{eq}); ^{13}C-NMR (75 MHz, $CDCl_3$); 140.6, 138.5, 138.1, 138.1, 128.5, 128.4, 128.3, 128.2, 128.0, 127.9, 127.9, 127.8, 127.8, 127.0, 127.6, 125.7 (ArC), 84.8 (C-1), 83.7 ($Ph\underline{C}H$), 80.4, 80.4, 79.8, 75.6 (C-2, C-3, C-4, C-5), 75.6, 75.2, 73.6 (OCH_2Ph), 68.8 (C-6), 35.5 (SCH_2); HRMS: Found $[M+Na]^+$ 591.2165, $C_{35}H_{36}O_5SNa$ requires 591.2176.

1,3,4,6-Tetra-*O*-acetyl-2-*O*-[1-methoxy-1-(*S*)-phenyl-2-(phenylsulfanyl)-ethyl]-α-D-glucopyranose (19)

Lead tetraacetate (47 mg, 0.105 mmol) in CH_2Cl_2 (300 µL) was added dropwise to a solution of 2-methoxy-2-(*S*)-phenyl-(3,4,6-tri-*O*-acetyl-1,2-dideoxy-β-D-gluco-pyranoso)[1,2-*e*]-1,4-oxathiane **14** (40 mg, 88 µmol) and 1-aminobenzotriazole (14 mg, 0.105 mmol) in CH_2Cl_2 (700 µL), at −78 ºC. After 30 min the temp was raised to −30 ºC, and held for 35 min before raising to r.t. After a further 1h 50 min the reaction mixture was diluted with CH_2Cl_2 (5 mL), washed with aq. $NaHCO_3$ (2 x 5 mL) and aq. NaCl (2 x 5 mL), dried (Na_2SO_4) and concentrated to afford a colourless syrup. The crude syrup was purified by flash chromatography (silica; 5:1 (v/v) hexane-ethyl acetate) to afford **19** (45 mg, 82%) as a colourless syrup; $[\alpha]_D^{23}$ +81.5 (*c* 0.5, $CHCl_3$); R_F 0.66 (3:2 (v/v) hexane-ethyl acetate); IR (ν_{max}/cm^{-1}): 2918 (C-H), 1751 (C=O); ^{1}H-NMR (500 MHz, $CDCl_3$); 7.47-7.10 (m, 10H, ArH), 6.31 (d, 1H, $J_{1,2}$ 3.4 Hz, H-1), 5.46 (dd, 1H, $J_{2,3}$ 9.7 Hz, $J_{3,4}$ 9.7 Hz, H-3), 4.88 (dd, 1H, $J_{3,4}$ 9.7 Hz, $J_{4,5}$ 9.6 Hz, H-4), 4.20 (dd, 1H, $J_{6,6'}$ 12.4 Hz, $J_{5,6}$ 4.4 Hz, H-6), 3.97-3.93 (m, 2H, H-5, H-6'), 3.78 (d, 1H, $J_{2,3}$ 9.7 Hz, $J_{1,2}$ 3.4 Hz, H-2), 3.57 (d, 1H, $J_{SCH2,SCH2'}$ 13.1 Hz, SCH_2), 3.39 (dd, 1H, $J_{SCH2,SCH2'}$ 13.1 Hz, SCH'_2), 3.31 (s, 3H, OCH_3), 2.22 (s, 3H, $C(O)CH_3$), 2.04 (s, 3H, $C(O)CH_3$), 2.03 (s, 3H, $C(O)CH_3$), 2.00 (s, 3H, $C(O)CH_3$); ^{13}C-NMR (75 MHz, $CDCl_3$); 170.6, 170.1, 169.5, 169.2 (C=O), 137.7, 135.9, 129.8, 129.7, 128.9, 128.8, 128.7, 128.7, 128.2, 127.4, 126.2 (ArC), 104.2 (C-OMe), 91.0 (C-1), 70.9 (C-3), 69.8, 68.7, 68.4 (C-2, C-4, C-5), 61.7 (C-6), 50.2 (OCH_3), 42.7 (SCH_2), 21.1, 20.9, 20.6, 20.5 ($C(O)\underline{C}H_3$); HRMS: Found $[M+Na]^+$ 613.1706, $C_{29}H_{34}O_{11}SNa$ requires 613.1714.

2-*O*-Acetyl-3,4,6-tetra-*O*-benzyl-α-D-glucopyranose (22) [2]

Lead tetraacetate (71 mg, 0.161 mmol) in CH_2Cl_2 (300 µL) was added dropwise to a solution of 2-methoxy-2-(*S*)-phenyl-(3,4,6-tri-*O*-benzyl-1,2-dideoxy-β-D-glucopyranoso)[1,2-*e*]-1,4-oxathiane **15** (80 mg, 0.134 mmol), 1-aminobenzotriazole (21.5 mg, 0.161 mmol) in CH_2Cl_2 (700 µL), at −78 ºC.

The reaction mixture was allowed to warm to r.t and stirred for 1 h 30 min, and then quenched with aq. $NaHCO_3$ (5 mL), and diluted with CH_2Cl_2 (5 mL). The organic layer was washed with aq. NaCl (5 mL), dried ($MgSO_4$) and concentrated to afford a colourless oil. The crude oil was purified by flash chromatography (silica; 7:1 (v/v) hexane-ethyl acetate→9:1 (v/v) CH_2Cl_2-methanol) to afford **22** (46 mg, 70%) as a colourless solid; m.p. 120-123 ºC, lit.[2] m.p. 124-126 °C; $[\alpha]_D^{21}$ +48.8 (*c* 0.8, $CHCl_3$), lit.[2]$[\alpha]_D^{23}$ +64 (*c* 1, $CHCl_3$); R_F 0.40 (2:1 (v/v) hexane-ethyl acetate); IR (ν_{max}/cm^{-1}): 3478 (OH), 1748 (C=O); ^{1}H-NMR (500 MHz, $CDCl_3$); 7.35-7.14 (m, 15H, ArH), 5.39 (d, 1H, $J_{1,2}$ 3.6 Hz, H-1), 4.87 (dd, 1H, $J_{2,3}$ 10.0 Hz, $J_{1,2}$ 3.6 Hz, H-2), 4.83-4.48 (m, 6H, 3 x OC$\underline{H}_2$Ph), 4.09-4.03 (m, 2H, H-3, H-4), 3.71-3.59 (m, 3H, H-5, H-6, H-6'), 3.55-3.41 (br d, 1H, OH), 2.02 (s, 3H, C(O)CH_3); m/z (ES+, %); 510.5 ($[M+NH_4]^+$);

1,3,4,6-Tetra-*O*-acetyl-2-*O*-[1-(*S*)-phenyl-2-(phenylsulfanyl)-ethyl]-α-D-glucopyranose (23) [3]

Lead tetraacetate (125 mg, 0.28 mmol) in CH_2Cl_2 (500 μL) was added dropwise to a solution of 2-(*S*)-phenyl-(3,4,6-tri-*O*-acetyl-1,2-dideoxy-β-D-glucopyranoso)[1,2-*e*]-1,4-oxathiane **17** (100 mg, 0.24 mmol), 1-aminobenzotriazole (38 mg, 0.28 mmol) in CH_2Cl_2 (1.5 mL), at −78 ºC. After 10 min the reaction mixture was quenched with aq. $NaHCO_3$ (5 mL), warmed to r.t. and diluted with CH_2Cl_2 (5 mL). The organic layer was washed with aq. NaCl (2 x 5 mL), dried ($MgSO_4$) and concentrated to afford a colourless oil. The crude oil was purified by flash chromatography (silica; 3:2 (v/v) hexane-ethyl acetate) to afford **23** (82 mg, 62%) as a colourless syrup; $[\alpha]_D^{23}$ +70.6 (*c* 2, $CHCl_3$), lit.[3] $[\alpha]_D^{20}$ +124.6 (*c* 0.6, $CHCl_3$); R_F 0.39 (2:1 (v/v) hexane-ethyl acetate); IR (ν_{max}/cm^{-1}): 2925 (C-H), 1754 (C=O); ^{1}H-NMR (500 MHz, $CDCl_3$); 7.35-7.16 (m, 10H, ArH), 6.47 (d, 1H, $J_{1,2}$ 3.5 Hz, H-1), 5.39 (dd, 1H, $J_{2,3}$ 9.7 Hz, $J_{3,4}$ 9.7 Hz, H-3), 4.90 (dd, 1H, $J_{3,4}$ 9.7 Hz, $J_{4,5}$ 9.7 Hz, H-4), 4.47 (dd, 1H, $J_{PhCH,SCH2}$ 8.4 Hz, $J_{PhCH,SCH2'}$ 4.5 Hz, PhCH), 4.25 (dd, 1H, $J_{6,6'}$ 12.4 Hz, $J_{5,6}$ 3.7 Hz, H-6), 4.04 (ddd, 1H, $J_{4,5}$ 9.7 Hz, $J_{5,6'}$ 3.7 Hz, $J_{5,6}$ 2.1 Hz, H-5), 3.99 (dd, 1H, $J_{6,6'}$ 12.4 Hz, $J_{5,6'}$ 2.1 Hz, H-6'), 3.58 (dd, 1H, $J_{2,3}$ 9.7 Hz, $J_{1,2}$ 3.5 Hz, H-2), 3.22 (dd, 1H, $J_{SCH2,SCH2'}$ 14.1 Hz, $J_{PhCH,SCH2}$ 8.4 Hz, SCH_2), 3.04 (dd, 1H, $J_{SCH2,SCH2'}$ 14.1 Hz, $J_{PhCH,SCH2'}$ 4.5 Hz, SCH'$_2$), 2.19 (s, 3H, C(O)CH_3), 2.03 (s, 3H,

$C(O)CH_3$), 1.98 (s, 3H, $C(O)CH_3$), 1.82 (s, 3H, $C(O)CH_3$); ^{13}C-NMR (75 MHz, $CDCl_3$); 170.5, 170.0, 169.5, 169.2 (C=O), 139.7, 136.3, 129.2, 129.0, 128.6, 126.9, 126.1 (ArC), 88.6 (C-1), 81.4 (C-2), 74.6 (Ph<u>C</u>H), 71.1 (C-5), 69.3 (C-3), 68.0 (C-4), 61.5 (C-6), 41.5 (SCH_2), 21.1, 20.6, 20.6, 20.5 (C(O)<u>C</u>H_3); m/z (ES+, %); 578.2 ($[M+NH_4]^+$, 5).

1-*O*-Acetyl-3,4,6-tetra-*O*-benzyl-2-*O*-[1-(*S*)-phenyl-2-(phenylsulfanyl)-ethyl]-α-D-glucopyranose (25)

Lead tetraacetate (70 mg, 0.16 mmol) in CH_2Cl_2 (300 μL) was added dropwise to a solution of 2-(*S*)-phenyl-(3,4,6-tri-*O*-benzyl-1,2-dideoxy-β-D-glucopyranoso)[1,2-*e*]-1,4-oxathiane **18** (75 mg, 0.13 mmol), 1-aminobenzotriazole (21 mg, 0.16 mmol) in CH_2Cl_2 (700 μL), at −78 ºC. After 10 min the reaction mixture was quenched with aq. $NaHCO_3$ (5 mL), warmed to r.t. and diluted with CH_2Cl_2 (5 mL). The organic layer was washed with aq. NaCl (2 x 5 mL), dried ($MgSO_4$) and concentrated to afford a colourless oil. The crude oil was purified by flash chromatography (silica; 7:1 (v/v) hexane-ethyl acetate) to afford **25** (53 mg, 57%, α:β: 96:4) as a colourless syrup; IR (ν_{max}/cm^{-1}): 2921 (C-H), 1750 (C=O); ^{1}H-NMR (500 MHz, $CDCl_3$); 7.35-7.06 (m, 25H, ArH), 6.44 (d, 1H, $J_{1,2}$ 3.2 Hz, H-1), 4.96 (d, 1H, J 11.1 Hz, OC<u>H</u>$_2$Ph), 4.79 (d, 1H, J 11.1 Hz, OC<u>H</u>$_2$Ph), 4.75 (d, 1H, J 10.4 Hz, OC<u>H</u>$_2$Ph), 4.66 (dd, 1H, $J_{PhCH,SCH2}$ 8.1 Hz, $J_{PhCH,SCH2'}$ 4.6 Hz, PhCH), 4.55 (d, 1H, J 12.2 Hz, OC<u>H</u>$_2$Ph), 4.43 (d, 1H, J 12.2 Hz, OC<u>H</u>$_2$Ph), 4.41 (d, 1H, J 10.4 Hz, OC<u>H</u>$_2$Ph), 3.93 (dd, 1H, $J_{3,4}$ 9.6 Hz, $J_{4,5}$ 9.6 Hz, H-4), 3.82 (dd, 1H, $J_{2,3}$ 10.6 Hz, $J_{1,2}$ 3.2 Hz, H-2), 3.70 (dd, 1H, $J_{6,6'}$ 11 Hz, $J_{5,6}$ 3.1 Hz, H-6), 3.63-3.56 (m, 3H, H-3, H-5, H-6'), 3.27 (dd, 1H, $J_{SCH2,SCH2'}$ 13.6 Hz, $J_{PhCH,SCH2}$ 8.1 Hz, SCH_2), 3.09 (dd, 1H, $J_{SCH2,SCH2'}$ 13.6 Hz, $J_{PhCH,SCH2'}$ 4.6 Hz, SCH'_2), 2.18 (s, 3H, $C(O)CH_3$); ^{13}C-NMR (75 MHz, $CDCl_3$); 170.0 (C=O), 139.7, 139.3, 138.5, 138.2, 137.5, 129.8, 129.3, 129.0, 128.8, 128.7, 128.4, 128.3, 128.1, 128.0, 127.8, 127.6, 126.4 (ArC), 89.4 (C-1), 81.5 (Ph<u>C</u>H), 79.9 (C-4), 77.3 (C-3), 76.1 (C-2), 75.9, 75.6, 74.0 (O<u>C</u>H_2Ph), 73.1 (C-5), 68.5 (C-6), 42.6 (SCH_2), 21.7 (C(O)<u>C</u>H_3); HRMS: Found $[M+Na]^+$ 727.2679, $C_{43}H_{44}O_7SNa$ requires 727.2700.

References

1. Armarego, W. L. F.; Perrin, D. D. *Purification of Laboratory Chemicals.* 4th ed.; Butterworth-Heinemann, 1996.
2. Schmidt, R. R.; Effenberger, G. *Carbohydr. Res.* **1987,** *171,* 59–79. doi:10.1016/S0008-6215(00)90879-6
3. Kim, J. H.; Yang, H.; Park, J.; Boons, G. J. *J. Am. Chem. Soc.* **2005,** *127,* 12090–12097. doi:10.1021/ja052548h
